# The diagnostic performance of endobronchial ultrasound with Xpert MTB/RIF Ultra in smear-negative pulmonary tuberculosis

**DOI:** 10.1186/s12879-023-08073-7

**Published:** 2023-02-22

**Authors:** Lan Yao, Shanhao Chen, Wei Sha, Ye Gu

**Affiliations:** 1 Shanghai Key Laboratory of Tuberculosis, Shanghai Pulmonary Hospital, Clinic and Research Center of Tuberculosis, Tongji University School of Medicine, Shanghai, 200433 China; 2 Department of Bronchoscopy, Shanghai Pulmonary Hospital, Tongji University School of Medicine, Shanghai, 200433 China

**Keywords:** Pulmonary tuberculosis, Smear-negative, Bronchoscopy, Xpert MTB/RIF Ultra

## Abstract

**Objective:**

This study investigated the diagnostic performance of endobronchial ultrasound with Xpert MTB/RIF Ultra (Ultra) for detecting smear-negative pulmonary tuberculosis (TB).

**Methods:**

143 patients suspected of sputum smear-negative pulmonary tuberculosis were enrolled in this study in Shanghai Pulmonary Hospital, China. These patients underwent endobronchial ultrasound with a guide sheath (EBUS-GS) or endobronchial ultrasound-guided transbronchial needle aspiration (EBUS-TBNA) based on their chest CT manifestations. We assessed the sensitivity and specificity of tissue specimens with Ultra in the TB group and non-TB group. Culture and clinical diagnosis were used as gold-standard for TB.

**Results:**

Among these 143 patients, 11 patients were culture-positive TB, 85 patients were diagnosed with culture-negative TB and 47 were with the non-TB diseases. Direct testing with microscopy (Acid-Fast Bacilli smear, AFB), liquid culture, pathology, Xpert MTB/RIF(Xpert) test and Ultra had a sensitivity of 8.3%, 11.5%, 42.7%, 64.6%, and 78.1% individually among all the TB patients. Ultra had a higher sensitivity than Xpert (*P* = 0.011). But Ultra had a specificity of 59.6% (95% CI 44.3–73.3), lower than that of Xpert (89.4%, 95% CI 76.1–96.0, *P* = 0.001). Ultra had the same sensitivity on specimens from EBUS-TBNA and EBUS-GS (*P* = 0.975). Ultra’s positive predictive value and negative predictive value were 79.8% and 57.1% respectively.

**Conclusions:**

Tissue specimens from interventional bronchoscopy combined with Ultra provide a sensitive method for diagnosing smear-negative pulmonary tuberculosis, but its specificity was lower than Xpert.

**Supplementary Information:**

The online version contains supplementary material available at 10.1186/s12879-023-08073-7.

## Background

As early as 2011, World Health Organization (WHO) issued a policy recommendation on using the Xpert MTB/RIF assay [1]. Xpert MTB/RIF was recommended by WHO as an initial diagnostic test in all adults suspected of having TB rather than conventional microscopy and culture in 2013 [2]. This test has greatly improved the diagnosis of tuberculosis globally. To further improve its sensitivity, Xpert MTB/RIF Ultra incorporates two different multi-copy amplification targets (IS6110 and IS1081) and has a larger DNA reaction chamber. Thus the limit of detection of tuberculosis reduces from 114 bacterial colony forming units (cfu) for Xpert MTB/RIF to 16 cfu per ml for Xpert MTB/RIF Ultra [3]. This is especially beneficial to smear-negative culture-positive tuberculosis patients as Ultra achieved the highest sensitivity increase in this group (+ 17%, 95% CI + 10, + 25) [3].

The bacteriologically confirmed pulmonary TB rate was 58% in China, lower than the average rate of 63% worldwide in 2021 [4]. However, more than one third of suspected TB patients cannot produce sufficient sputum for diagnosis [5]. Endobronchial ultrasound with biopsy techniques like EBUS-GS [6] and EBUS-TBNA [7] can play an important role in the collection of enough tissue specimens for TB diagnosis.

In this study, we combined the endobronchial ultrasound methods with Xpert MTB/RIF Ultra to evaluate their detection ability in smear-negative pulmonary tuberculosis (PTB).

## Methods

### Study population

This was a single-center, prospective, diagnostic accuracy study. Participants were enrolled from September 2018 to December 2019 in Shanghai Pulmonary Hospital, one of the top tuberculosis hospitals in China. Inclusion criteria: (1) Suspicion of pulmonary tuberculosis (with symptoms and radiological examination outcomes); (2) No contraindication for bronchoscopy; (3) Sputum smear negative twice; (4) Chest CT showed pulmonary lesions that could take tissue specimens by EBUS-TBNA or EBUS-GS; (5) HIV negative.

### Bronchoscopy sampling

Patients with bronchial signs in the lesions were treated with EBUS-GS. They underwent bronchoscope to access the lesion via the planned path. After the lesion site was confirmed by an ultrasound probe through the guide sheath, the probe was removed. A forcep was inserted through the guide sheath to collect enough specimens. Then we brushed for the AFB test. EBUS-TBNA was performed on patients who had enlargement of mediastinal or hilar lymph nodes or centrally located peribronchial lesions. We used a curvilinear ultrasound bronchoscope (Olympus BF-UC 260 FW) to locate the lesion or lymph node and then punctured three times with a 22 gauge needle to aspirate enough specimens.

### AFB, culture, Xpert, Ultra and pathological examination

Both pathological and microbiological (AFB smear, culture, Xpert MTB/RIF and Xpert MTB/RIF Ultra) tests were conducted. Each biopsy specimen was fixed in formalin, embedded, and stained with hematoxylin and eosin for pathological examination. Brushing and aspiration smears were prepared and stained with the Ziehl–Neelsen method [6]. The results were graded as negative or positive (scanty, 1+, 2+, 3+, 4+). The rest of the samples were ground and homogenized with PBS buffer. After centrifugation and resuspended the sediments in 2 ml PBS buffer via vortexing. Part of the specimens was used for liquid culture (BACTEC MGIT, Becton Dickinson, Cockeysville, MD, USA). The supernatant of the culture-positive strain was tested by MPT64 antigen detection (SD Bioline Kit, Standard Diagnostics, Korea) [8] to rule out nontuberculous mycobacteria (NTM) infection. The remaining specimens were used for Xpert MTB/RIF (Cepheid, USA) [9] and Xpert MTB/RIF Ultra (Cepheid, USA) [10] according to the manufacturer’s instructions. GeneXpert sample reagent was added to the remaining specimen, vortexed and incubated at room temperature for 15 min. Then the digested sample was transferred to one Xpert and one Ultra cartridge and loaded onto instruments. The results of the detection of MTB can be automatically generated by instruments.

### Diagnosis and grouping

Culture and clinical diagnosis were used as gold-standard for TB. Patients were divided into three categories for analysis according to their diagnosis: those with culture-positive pulmonary tuberculosis (culture-positive TB); those with culture-negative pulmonary tuberculosis who had improvement with anti-tuberculous treatment based on clinical and radiologic findings (culture-negative TB); and those with no evidence of TB and diagnosed with other pulmonary diseases based on pathological and laboratory findings (non-TB). Smear-negative pulmonary tuberculosis was defined as two sputum smear negative. The histopathological diagnosis criteria for TB was granuloma with cheesy necrosis. Patients were all followed-up in the outpatient department to correct diagnosis.

### Statistical method

The median and range were calculated to determine the study population. The χ^2^ test, Fisher test, and t-test were used to verify whether there were significant differences between enrolled TB patients and non-TB patients. Two-sided *P* value < 0.05 was set as statistically significant. For binomial distribution data, we used 95% confidence intervals (95% CI) to report the number of positive samples and the corresponding proportion. All analysis and graphs were performed by SPSS® 26.0 (SPSS Inc., Chicago, IL, USA) and prism 7.0 software (Graphpad Software Inc., San Diego, CA, USA).

## Results

### Patients

**Fig. 1:**
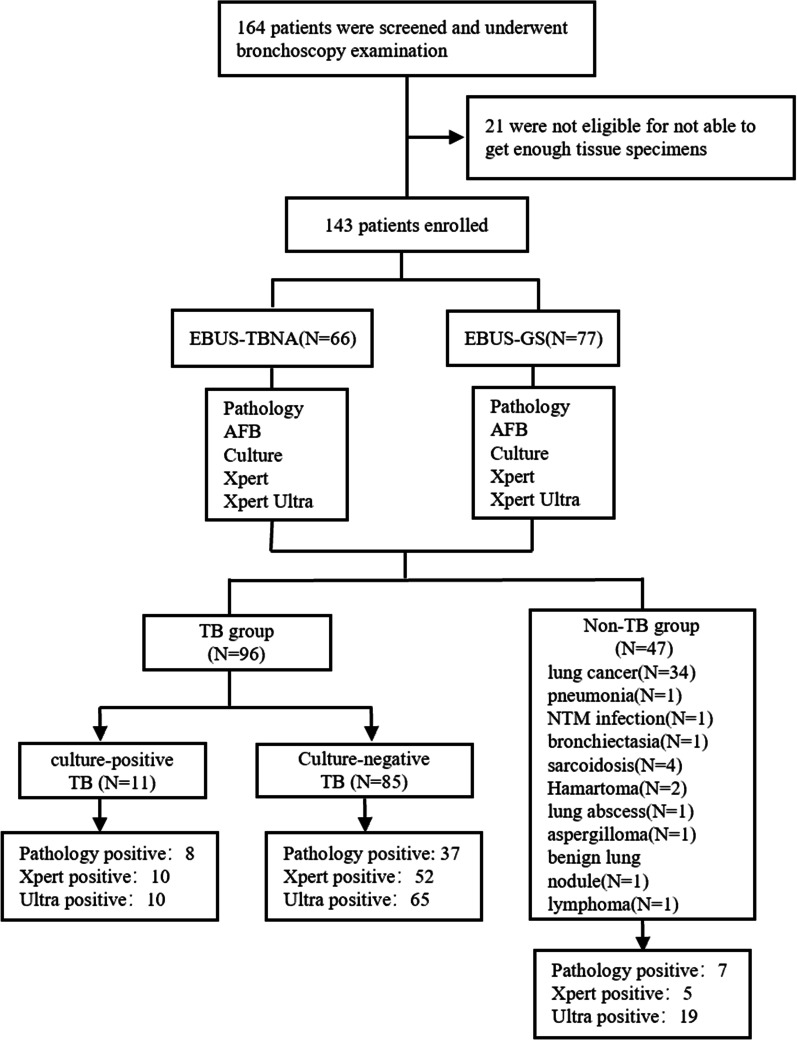
Flow diagram of the study population

A total of 164 smear-negative patients with suspected smear-negative pulmonary tuberculosis were screened in this study (Fig. 1). 21 were excluded for not able to get tissue specimens by EBUS-TBNA or EBUS-GS. Then 143 patients enrolled in the final analysis. Among these patients, 96 (67.1%) were diagnosed with pulmonary tuberculosis (TB group); In the TB group, 11 patients were culture-positive TB and 85 patients were diagnosed with culture-negative TB; 47 (32.9%) were in the non-TB group. The TB group had 50 (50.1%) patients undergo EBUS-TBNA while the non-TB group had 16 (34.0%) patients to do the same test.

The demographic characteristics of both groups were showed in Table 1. We collected demographic information of both groups, including: sex, age, tuberculosis history, T-SPOT.TB (T-SPOT) result, complication, and chest CT manifestation. Among all the enrolled patients, 56.6% were males. The age range was from 11 to 82 years old with a median of 45 years old. The TB group was younger than the non-TB group (33.5 vs 56, *P* = 0.000). The tuberculosis history and complication were no difference between these two groups. But the TB group had a higher rate of T-SPOT positive results (86.4% vs 22.5%, *P* = 0.000) and fever symptoms (24.0% vs 8.7%, *P* = 0.027). The differences were also seen in the imaging findings. The non-TB group tended to have lesions in single lobe (70.2% vs 46.9%, *P* = 0.008) and have less shadow in the CT scan (25.5% vs 74.0%, *P* = 0.000).

**Table 1:** Demographic characteristics of the TB group and non-TB group

Clinical characteristics	All patients (N = 143)	TB group (N = 96)	Non-TB group (N = 47)	*P*
Sex, male (no, %)	81 (56.6)	55 (57.3)	26 (55.3)	0.823
Age, median (range)	45 (11–82)	33.5 (16–82)	56 (11–74)	0.000
Had tuberculosis history	12 (8.4)	5 (5.2)	7 (14.9)	0.06
T-spot positive (no/total no, %)	85/126 (67.5)	76/86 (86.4)	9/40 (22.5)	0.000
Complication (no, %)				
Diabetes mellitus	5 (3.5)	4 (4.2)	1 (2.1)	1.000
Chronic respiratory diseases^a^	3 (2.1)	1 (1.0)	2 (4.3)	0.251
Symptoms (no, %)				
Cough	59 (41.3)	39 (40.6)	20 (42.6)	0.826
Hemoptysis	8 (5.6)	4 (4.2)	4 (8.5)	0.288
Fever	27 (18.9)	23 (24.0)	4 (8.7)	0.027
Range of lung lesions				0.008
In single lobe	78 (54.5)	45 (46.9)	33 (70.2)	
In multiple lobes	65 (45.5)	51 (53.1)	14 (30.0)	
Chest CT manifestation				
Nodule	113 (79.0)	74 (77.1)	39 (83.0)	0.416
Cavitation	11 (7.7)	7 (7.3)	4 (8.5)	0.797
Shadow	83 (58.0)	71 (74.0)	12 (25.5)	0.000

^a^Chronic respiratory diseases included chronic bronchitis, asthma, and chronic obstructive pulmonary disease

The common side effects of bronchoscopy are airway bleeding and fever which occurred in 12 (8.4%) cases and 3 (2.1%) cases separately.

### Diagnostic performance of AFB, culture, pathology, Xpert and Ultra

Among 11 culture-positive TB, Ultra detected positive results in 10 cases (90.9%), the same sensitivity as Xpert. Among all the 96 smear-negative TB patients, EBUS-TBNA and EBUS-GS combined had reached an AFB positive rate of 8.3% (8/96) and a culture positive rate of 11.5% (11/96) (Table 2). The pathological diagnosis provided by tissue specimens had a higher positive rate of 42.7%, and its specificity was 85.1%. The Xpert positive rate on the tissue specimen was 64.6%, and its specificity was 89.4%. Ultra had the highest sensitivity of 78.1% among these methods, statistically higher than Xpert (*P* = 0.011). But its specificity was the lowest (59.6%), statistically lower than Xpert (*P* = 0.001). Ultra’s positive predictive value and negative predictive value were 79.8% and 57.1% respectively.

**Table 2:** The diagnostic performance of AFB, culture, pathological examination, Xpert and Ultra, using culture or final diagnosis as gold-standard

	Method	% (correct no./total no.) 95% CI			
		Sensitivity	Specificity	PPV	NPV
For culture-positive TB patients (n = 11)	AFB	63.6 (7/11) 31.6–87.6	100 (47/47) 90.6–100	100 (7/7) 56.1–100	92.2 (47/51) 80.3–97.5
	Pathology	72.7 (8/11) 39.3–92.7	85.1 (40/47) 71.1–93.3	53.3 (8/15) 27.4–77.8	93.0 (40/43) 79.9–98.2
	Xpert	90.9 (10/11) 57.1–99.5	89.4 (42/47) 76.1–96.0	66.7 (10/15) 38.7–87.0	97.7 (42/43) 86.2–99.9
	Ultra	90.9 (10/11) 57.1–99.5	59.6 (28/47) 44.3–73.3	34.5 (10/29) 18.6–54.3	96.6 (28/29) 80.4–99.8
For culture-positive and negative TB patients (n = 96)	AFB	8.3 (8/96) 3.9–16.2	100 (47/47) 90.6–100	100 (8/8) 59.8–100	34.8 (47/135) 27.0–43.5
	Culture	11.5 (11/96) 6.1–20.0	100 (47/47) 90.6–100	100 (11/11) 67.9–100	35.6 (47/132) 27.6–44.5
	Pathology	42.7 (41/96) 32.8–53.2	85.1 (40/47) 71.1–93.3	85.4 (41/48) 72.8–92.8	42.1 (40/95) 32.2–52.7
	Xpert	64.6 (62/96)^a^ 54.1–73.9	89.4 (42/47) 76.1–96.0	92.5 (62/67) 86.1–99.0	55.3 (42/76) 43.8–66.7
	Ultra	78.1 (75/96)^a^ 68.3–85.7	59.6 (28/47) 44.3–73.3	79.8 (75/94) 71.5–88.1	57.1 (28/49) 42.8–71.5

*SE* sensitivity, *SP* specificity, *PPV* positive predictive value, *NPV* negative predictive value

^a^McNemar test *P* = 0.011 (Ultra vs. Xpert)

**Fig. 2:**
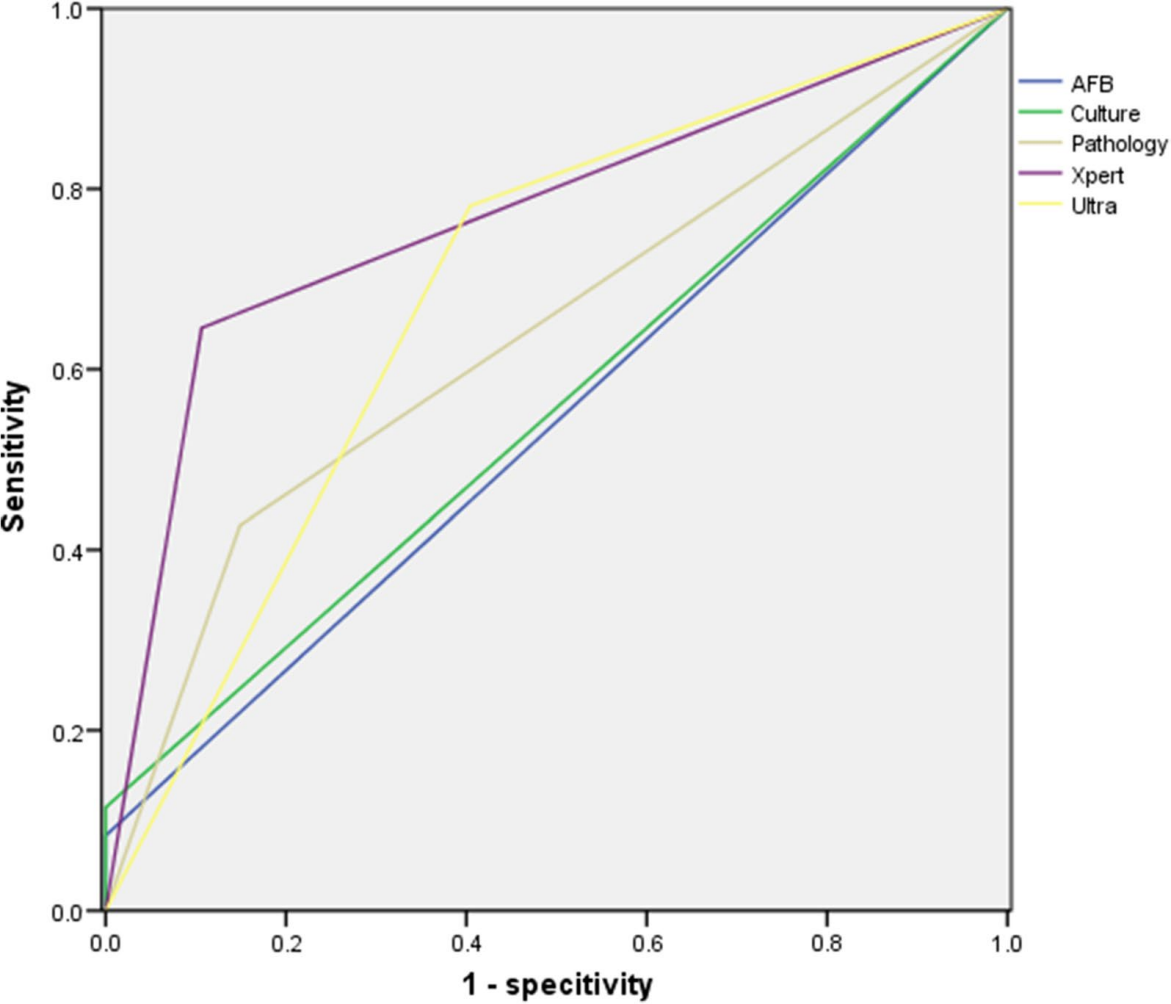
Receiver operating characteristic (ROC) curves of AFB, culture, pathology, Xpert and Ultra on tissue specimens

The corresponding receiver operating characteristic (ROC) curves for AFB, culture, pathology, Xpert and Ultra on tissue specimen are shown in Fig. 2. The areas under the curve (AUC) for AFB, culture, pathology, Xpert and Ultra were 0.542, 0.557, 0.639, 0.770 and 0.688.

### Comparison of detection rates among specimens from EBUS-TBNA and EBUS-GS by Ultra

There was no significant difference in the diagnostic performance of Xpert Ultra tested on specimens from different methods (*P* = 0.975). Ultra had a detection rate of 78.0% (23/50) on EBUS-TBNA specimens and 78.3% (18/46) on EBUS-GS specimens, respectively. The detection rate of Ultra on EBUS-TBNA specimens and EBUS-GS specimens were both higher than those of Xpert, but they were not statistically significant (Additional file 1: Table S1).

## Discussion

Diagnosis of smear-negative pulmonary tuberculosis is a challenge for society. With limited laboratory resources and understaffed situations, the diagnosis in low-income countries depends mostly on clinical and radiological indicators [11]. The most common radiographic manifestation of PTB is parenchymal opacities in the apical and posterior segments of the upper lobes and the superior segments of the lower lobes, usually more than one segment [12]. The most common CT manifestation is the bronchogenic spread of infection (tree-in-bud), which can be identified in 95% of pulmonary TB [13]. Single or multiple cavitations, the radiological hallmark of PTB, can be found in 40% to 45% cases [12, 13]. But some atypical CT manifestations like intrathoracic lymphadenopathy, mediastinal lesions, single pulmonary nodule or unconventional lesion site of pulmonary tuberculosis also increase difficulties of diagnosis for clinicians. An atypical distribution of parenchymal opacities in uncommon segments accounts for approximately 5% of cases [13]. Hilar and mediastinal lymphadenopathy also occur in approximately 5% of cases [13]. The atypical CT manifestations occur more frequently in people living with HIV and other immunosuppressed populations like patients with diabetes mellitus and the elderly. They may be misdiagnosed or missed diagnosed for lack of effective diagnostic methods.

In this situation, the pathological examination is quite important in the field of differential diagnosis. EBUS-GS with a flexible catheter guided by ultrasound probe is helpful and has minimal invasive in accessing the tissue specimens with reachable bronchial signs in the lesions [6, 14]. Among patients with intrathoracic lymphadenopathy, EBUS-TBNA is a technique that allows direct sampling to increase the sensitivity and culture yield of tuberculosis [15–17]. Cytopathological or pathological findings alone revealed TB in 72.7% [15] to 84.2% of patients [17]. So compared with smear and culture, pathological diagnosis is quite sensitive but its specificity was lower in our study. Granulomatous inflammation can be found in many other diseases like sarcoidosis, nontuberculous mycobacterial disease, parasites, fungi, autoimmune disease, and even lymphoma [18]. So pathological examination alone may cause a false positive result.

Tissue samples with bacteriological examinations have some advantages over simply pathological diagnosis. Xpert MTB/RIF Ultra has 5% higher sensitivity than Xpert MTB/RIF [3], and it has been recommended as an initial diagnostic test in adults with signs and symptoms of pulmonary TB and without a prior history of TB (≤ 5 years) or with a remote history of TB treatment (> 5 years since end of treatment) or with a prior history of TB and an end of treatment within the last 5 years in the latest WHO policy [19]. Ultra has already been applied to diagnose extrapulmonary tuberculosis. In the diagnosis of pleural TB, Ultra using pleural fluids is better than Xpert [20, 21], but using biopsy samples maybe even better [22–24]. One study combining pathological examination for biopsy with Ultra increased the sensitivity to 92.59% (25/27) [25]. It is the same on other types of tissue samples, like lymph node aspirate or lymph node biopsy [21, 26] and bone or joint aspirate [27, 28]. However, the increased sensitivity of Ultra reduces the overall specificity in high-burden TB settings for detecting of dead bacilli in patients with recent history of TB [3]. In this study, the non-TB group had a 14.9% of tuberculosis history and 22.5% of T-spot positive rate, which may contribute to the low specificity of Ultra.

## Limitation

The limitation of this study was the relatively small study population and the uneven distribution of enrolled patients in different groups. The non-TB group had a smaller size compared with the TB group. And the patients underwent EBUS-TBNA were less than half the number of patients underwent EBUS-GS, which made tissue specimens mainly come from EBUS-GS. So we need larger studies to further evaluate the performance of Xpert Ultra with tissue specimens from interventional bronchoscopy.

## Conclusion

In conclusion, tissue specimens from interventional bronchoscopy combined with Xpert MTB/RIF Ultra could provide a sensitive method for diagnosing smear-negative pulmonary tuberculosis, but its specificity was lower than Xpert MTB/RIF.

## Supplementary Information

**Additional file 1: Table S1.** The detection rate of culture, pathology, Xpert and Ultra on specimens from EBUS-TBNA and EBUS-GS.

## Data Availability

All data analyzed in this study are included in this published article.
