# Supplementary material for: The diagnostic performance of endobronchial ultrasound with Xpert MTB/RIF Ultra in smear-negative pulmonary tuberculosis
Source: BMC Infect Dis. 2023 Feb 22;23:107. doi: 10.1186/s12879-023-08073-7 (PMC9945607; doi:10.1186/s12879-023-08073-7)
Supplement: Supplementary file 1 — Additional file 1: Table S1. The detection rate of culture, pathology, Xpert and Ultra on specimens from EBUS-TBNA and EBUS-GS. [file 12879_2023_8073_MOESM1_ESM.docx]

Table S1. The detection rate of culture, pathology, Xpert and Ultra on specimens from EBUS-TBNA and EBUS-GS.

| Method | EBUS-TBNA  Sensitivity (%, 95% CI)  N=50 | EBUS-GS  Sensitivity (%, 95% CI)  N=46 |
| --- | --- | --- |
| Culture | 10.0(1.4-18.6) | 13.0(2.9-23.2) |
| Pathology | 46.0(31.7-60.3) | 39.1(24.5-53.8) |
| Xpert | 68.0(54.6-81.4)^a^ | 60.9(46.2-75.5)^b^ |
| Ultra | 78.0(66.1-89.9)^a^ | 78.3(65.9-90.6)^b^ |

a: McNemar Test *P*=0.180 (Ultra vs. Xpert)

b: McNemar Test *P*=0.057 (Ultra vs. Xpert)
